# Can an incentive-based intervention increase physical activity and reduce sitting among adults? the ACHIEVE (Active Choices IncEntiVE) feasibility study

**DOI:** 10.1186/s12966-017-0490-2

**Published:** 2017-03-21

**Authors:** Kylie Ball, Ruth F. Hunter, Jaimie-Lee Maple, Marj Moodie, Jo Salmon, Kok-Leong Ong, Lena D. Stephens, Michelle Jackson, David Crawford

**Affiliations:** 1 Institute for Physical Activity and Nutrition (IPAN), School of Exercise and Nutrition Science, Deakin University, Geelong, Australia; 2 UKCRC Centre of Excellence for Public Health Research Northern Ireland, Queen’s University Belfast, Belfast, Northern Ireland UK; 3 Deakin Health Economics, Centre for Population Health Research, Faculty of Health, Deakin University, Geelong, Australia; 4 La Trobe Business School, La Trobe University, Bundoora, VIC Australia; 5 Institute for Physical Activity and Nutrition, Deakin University, 221 Burwood Highway, Burwood, 3125 VIC Australia

**Keywords:** Physical activity, Sedentary behavior, Intervention, Incentivisation, Contingency management theory, Control theory, Body mass index, Blood pressure

## Abstract

**Background:**

Despite recent interest in the potential of incentivisation as a strategy for motivating healthier behaviors, little remains known about the effectiveness of incentives in promoting physical activity and reducing sedentary behavior, and improving associated health outcomes.

This pre-post-test design study investigated the feasibility, appeal and effects of providing non-financial incentives for promoting increased physical activity, reduced sedentary time, and reduced body mass index (BMI) and blood pressure among inactive middle-aged adults.

**Methods:**

Inactive men (*n* = 36) and women (*n* = 46) aged 40–65 years were recruited via a not-for-profit insurance fund and participated in a 4 month pre-post design intervention. Baseline and post-intervention data were collected on self-reported physical activity and sitting time (IPAQ-Long), BMI and blood pressure. Participants were encouraged to increase physical activity to 150 mins/week and reduce sedentary behavior by 150 mins/week in progressive increments. Incentives included clothing, recipe books, store gift vouchers, and a chance to win one of four Apple iPad Mini devices. The incentive component of the intervention was supported by an initial motivational interview and text messaging to encourage participants and provide strategies to increase physical activity and reduce sedentary behaviors.

**Results:**

Only two participants withdrew during the program, demonstrating the feasibility of recruiting and retaining inactive middle-aged participants. While two-thirds of the sample qualified for the easiest physical activity incentive (by demonstrating 100 mins physical activity/week or 100 mins reduced sitting time/week), only one third qualified for the most challenging incentive. Goals to reduce sitting appeared more challenging, with 43% of participants qualifying for the first incentive, but only 20% for the last incentive. More men than women qualified for most incentives. Mean leisure-time physical activity increased by 252 mins/week (leisure-time), with 65% of the sample achieving at least 150 mins/week; and sitting time decreased by 3.1 h/day (both *p* < 0.001) between baseline and follow-up. BMI, systolic and diastolic (men only) blood pressure all significantly decreased. Most participants (50–85%) reported finding the incentives and other program components helpful/motivating.

**Conclusions:**

Acknowledging the uncontrolled design, the large pre-post changes in behavioral and health-related outcomes suggest that the ACHIEVE incentives-based behavior change program represents a promising approach for promoting physical activity and reducing sitting, and should be tested in a randomized controlled trial.

**Trial registration:**

Australian New Zealand Clinical Trials Registry IDACTRN12616000158460, registered 10/2/16.

## Background

Physical inactivity and sedentary lifestyles are major contributors to disease burden, increasing the risk of a range of adverse health outcomes including cardiovascular events, type 2 diabetes, depression and mortality [1]. In the context of epidemic rates of sedentariness and obesity, there is increasing interest in using incentives to motivate changes in health behaviors, in an effort to foster a greater stake in improving health, encourage disease prevention, and reduce health burden and associated costs, across government, non-government organisations and the health insurance industry [2].

Incentives comprise financial or non-financial rewards for progressing towards or achieving targets in desired (or reducing undesired) health behaviors. Incentives are hypothesized by learning theory principles to provide an immediate reward for behaviors that confer long-term health benefits [3–5]. However, there remains a paucity of evidence of the effectiveness of these approaches for promoting behavior change. Findings suggest that even small incentives can influence physical activity behaviors, particularly among previously inactive participants [6]. However evidence of the effectiveness of incentives for promoting physical activity remains mixed [7, 8]. Studies remain relatively limited in number and scope, focusing mostly on structured exercise (e.g., gym or walking group attendance), rather than free-living lifestyle physical activity. Only one study was identified which evaluated the effects of incentives on reducing sedentary time [9]. That study allocated 204 participants (24% men) into one of four groups with varying target healthy behavior change combinations (increase fruit and vegetable consumption, increase physical activity, decrease fat, decrease sedentary behavior during leisure time). Participants could earn a $175 incentive for meeting goals for targeted behaviors. The reduction in sedentary leisure time combined with an increase in fruit and vegetable consumption was the target behavior combination with the most significant improvements. Sedentary leisure time in particular decreased from 219.2 min per day at baseline to 89.3 min per day post-treatment [9]. However, this intervention also comprised a relatively intensive individually-tailored behavioral coaching component, including daily goal-setting and coach communication, which is likely to have made a substantial contribution to the observed behavior change.

Given the paucity of evidence on the impact of incentives-based approaches for promoting physical activity and reducing sedentary behavior, little is known about the specific components of incentive-based approaches that might contribute to greater behavior change. Some research suggests that indexed and escalating incentives (e.g., a set and increasing value incentive awarded for each exercise class attended) may be more effective than non-indexed incentives in promoting behavior change [10]. There is also evidence that ‘assured’ incentives are more effective than those that are lottery-based [6], and that incentives provided soon after the achievement of the qualifying behavior may be more effective than those provided weeks or months later, given individuals’ tendencies to be more motivated by immediate than delayed gratification [11]. It has been suggested that for optimal effects, incentives should be embedded alongside other proven behavior change techniques, such as goal-setting, self-monitoring, and providing social support, in order to foster increased intrinsic motivation that is sustained after incentives cease [3, 12]. The use of non-financial or in-kind incentives such as goods provided by businesses, or incentives that tap into customer loyalty schemes, have been argued as preferable over cash payments, since the former may comprise a more sustainable business model [13]. However, there is a lack of evidence about the effectiveness or feasibility of such approaches.

This study investigated the feasibility and effects of providing non-financial incentives for promoting increased physical activity and reduced sedentary time among inactive middle-aged adults. Intervention effects on changes to body mass index (BMI) and blood pressure were also investigated.

## Methods

### Design and ethics

The ACHIEVE (Active Choices IncEntiVE) Study used an uncontrolled, pre-post-test design with a 4-month intervention period. This design was appropriate for establishing the feasibility, appeal and potential effectiveness of an innovative intervention prior to launching into a more costly and intensive randomized controlled trial design. A 4-month intervention period has been shown to be long enough for participants to develop new physical activity habits [14].

The intervention took place between June and November 2015 in Melbourne, Australia, and was approved by the Deakin University Faculty of Health Human Ethics Advisory Group (HEAG-H 179_2014). All participants provided written informed consent.

### Recruitment and participants

Incentivisation of healthy behaviors is of interest to health insurance bodies, and we partnered in this study with GMHBA Health Insurance, a leading not-for-profit health insurance fund in Victoria, Australia. While all Australian citizens and permanent residents are covered by Australia’s universal, publically funded, government operated health care scheme (Medicare), 55.8% of Australians also hold private health insurance [15], which provides additional benefits (e.g., subsidies for additional health services, increased choice of providers, shorter wait times for elective surgeries). Participants were recruited through the GMHBA member database, with GMHBA membership socio-demographically diverse.

The sampling frame comprised GMHBA members aged 40–65 years, as this life stage is characterized by declining levels of physical activity and increased risk of chronic disease onset [2, 16]. Adults were eligible if they lived within 25 km of the study site (for pragmatic reasons), did not meet current physical activity guidelines (self-report), and spent more than three quarters of their day sitting on most days (self-report). A sample of 80 was estimated to provide 80% power at α = 0.05 to detect an effect size in the primary outcome, an increase in physical activity duration, of at least 0.3 (equivalent to 60 min of physical activity/week) allowing for up to 10% attrition over the 4-month study period. While participants were encouraged to progress beyond this to achieve 150 mins/week physical activity, power calculations were based on a lower achievement threshold that was considered feasible but significant in this initially sedentary sample.

**Fig. 1:**
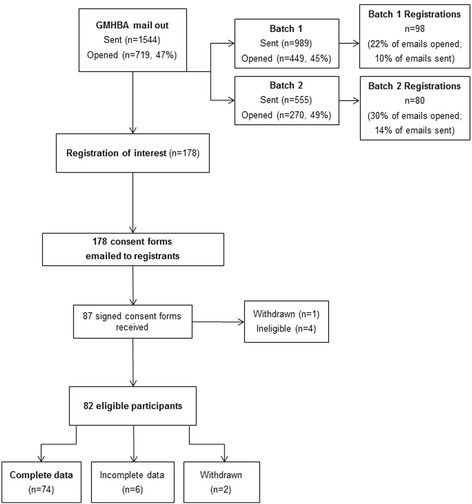
Participant recruitment and flow through the study

In May 2015, GMHBA sent an initial batch of study invitations by Electronic Direct Mail to 1544 potentially eligible members. Interested participants who self-screened as eligible were asked to register their interest in the study via a web-link. Participant recruitment and flow through the study is presented in Fig. 1. Registrations of interest were received from 178 members (25% of the 719 who viewed the email, or 11.5% of the targeted sample), within a week of the invitation being emailed. Upon receiving the registration, the research team emailed participants a plain language statement and consent form. One participant withdrew before beginning the study, and a further four participants were excluded (three exceeded physical activity guidelines and one could not attend a measurement appointment). Excluding two participants who withdrew during the study (due to time constraints and illness), 35 men and 45 women took part in the program.

### Intervention

The main intervention component was provision of non-financial incentives contingent on behavior change (increased physical activity and reduced sedentary time); however, consistent with recommendations and evidence-based behavior change theory [3, 17], incentives were incorporated into a broader suite of behavior change strategies designed to enhance self-efficacy and intrinsic motivation to be active. These included a motivational interview [18] and weekly text messages from the research team based on principles of Control Theory [19], which posits that behavioral self-regulation is enhanced by setting goals; monitoring behavior; receiving feedback and reviewing goals after feedback [17, 19].

Motivational interview: At intervention commencement, participants were telephoned at a pre-arranged time to take part in a motivational interview of approximately 20 min duration, conducted by one of two research staff. Meta-analytic evidence supports the effectiveness of a motivational interview for supporting behavior change [20]. During the interview participants were asked their views on the benefits they felt would be gained from increasing physical activity and reducing sedentary behaviors, and their confidence in doing so. The interviewer assisted the participant to generate tailored strategies to increase physical activity, particularly leisure-time and transport-related activity, and to reduce sitting time, particularly during leisure-time although sitting at work was also a focus. At the conclusion of the interview, participants were told they could begin aiming to achieve their target minutes of increased physical activity and reduced sedentary time immediately.

Incentives: The incentive approach is described here according to the framework recommended by Adams et al. [21]. The intervention rewarded positive behaviors (‘gain’ framed), encouraging participants to increase physical activity and reduce sedentary behavior in progressive increments, with the ultimate aim of achieving 150 min of physical activity per week, and a reduction of 150 min per week of sedentary time. Participants received daily points for participating in physical activity, capped at 30 min per day, with one point per minute allocated for engaging in at least moderate-intensity physical activity, and one point per minute reduction in sedentary behavior, with the overall goal of at least 30 mins/day of activity and 30 mins/day reduction in sedentary behaviors. When sufficient points were accrued (see incentives schedule in Table 1), participants were posted incentives at 2 weeks post-baseline, 4 weeks, and then monthly. The incentives schedule was designed to be incremental, consistent with Contingency Management theory, which posits that gradually increasing the value of incentives as behavior change progresses or is maintained will produce more sustained behavior change [21, 22]. The level of challenge of the physical activity and reduced sitting goals, and the value of incentives, hence increased over the course of the intervention. Incentives included clothing, recipe books, and store gift vouchers (‘certain’ incentives, guaranteed upon achieving the target physical activity/sitting reductions), collectively ranging in value from AUD$7.50 to $50 each (total value $193.50 for women and $196.50 for men); and a chance to win one of four Apple iPad Mini devices (‘uncertain’ lottery-based incentive), worth $454.

**Table 1:** ACHIEVE Incentives schedule and dollar value (AUD$)

	First 2 weeks	Second 2 weeks (Month 1)	Month 2	Month 3	Month 4
For increased physical activity: one point/minute, capped at 30 mins/day (total possible 210 points/week)	For achieving 200 PA points (100 mins PA/week) Women’s scarf, $7.50 Men’s cap $10.50	For achieving 200 PA points (100 mins PA/week) $10 Supermarket voucher	For achieving 240 PA points (120 mins PA/week) Heart Foundation cookbook, $17 or $20	For achieving 300 PA points (150 mins PA/ week) $50 supermarket voucher	For maintaining 300 PA points (150 mins PA/week) Chance to win one of four Apple iPad Minis
For reduced sitting time: one point/minute reduction from baseline, capped at 30 mins/day (total possible 210 points/week)	For achieving 200 SB points (100 mins reduction/week) $10 Supermarket voucher	For achieving 200 SB points (100 mins reduction/week) Heart Foundation shirt, $18	For achieving 240 SB points (120 mins reduction/week) $40 Supermarket voucher	For achieving 300 SB points (150 mins reduction/week) Heart Foundation hooded jacket, $38	For maintaining 300 SB points (150 mins reduction/week reduction from baseline) Chance to win one of four Apple iPad Minis
Total value for participants	$17.50 ($8.25/week) for women $20.50 (10.20/week) for men	$28.00 ($14/week)	Average ($14.3/week)	$88 ($22/week)	

Text Messaging: Mobile telephone short text messages were sent to participants once per week across the duration of the study to encourage them and provide strategies to increase physical activity and reduce sedentary behaviors. A library of text messages was developed, informed by principles of Control Theory [19], which is based on the premise that individuals seek feedback, and set goals based on that feedback. Control theory principles used in this intervention include self-regulation techniques to prompt goal setting and intention formation (e.g., ‘Do you know how you are going to be active tomorrow? Think ahead about how you will achieve your physical activity goals this week’; prompt self-monitoring of behavior (e.g., ‘Sync your FitBit at least 1 × week to keep track of your activity. You can also pop on the scales and email us your weight too. ACHIEVE team’), provide feedback on performance, and prompt review of behavioral goals (e.g., ‘Have you reviewed your physical activity & reduced sitting goals? Try setting a new goal to walk 30 mins more or sit 30 mins less today. ACHIEVE team’). Mid-way through the study, participants were contacted by text message to weigh themselves and email the results to the research team. Text messages were also sent for administrative purposes (e.g., reminder messages about the measurement appointment, taking blood pressure readings, or completing evaluation surveys). Immediately after the 16 week intervention participants provided final weight and blood pressure measures, and completed the online post-study survey.

### Procedure

Upon recruitment, researchers contacted participants to provide a link to the online pre-study survey and to arrange an initial appointment, either at Deakin University or the participant’s home or workplace, during which research staff measured height using a Seca 217 portable stadiometer, and weight using Wi-Fi digital scales. Two research assistants (both Masters-qualified), and two research fellows (PhD-qualified) were trained to deliver the interview component of the intervention. As participants were required to measure their own weight during the study, research staff demonstrated the correct way to measure their weight (i.e. remove shoes and heavy clothing, e.g., jackets; all parts of their feet on the scale, hands by sides and standing up straight). Research staff also provided a FitBit One (FitBit, Inc., San Francisco, CA, US), a commercially available clip-based personal activity monitor typically worn at the hip that tracks steps, distance, time spent sedentary and in physical activity of different intensities, calories burned, floors climbed and sleep (to facilitate self-monitoring and feedback). Physical activity and reduced sitting behavior were monitored via participants regularly uploading FitBit data. These data were linked to the ACHIEVE website, which automatically calculated daily and weekly points and notified participants if they qualified for incentives. Staff also provided a set of Wi-Fi digital scales to measure weight during the study; and a clinically validated [23]. Omron wrist-worn blood pressure monitor (Model HEM-6121), that was loaned to participants for 1 week at baseline and immediately post-intervention data collection points.

Participants were instructed on the use of all devices and on the ACHIEVE website designed for the study and linked to participants’ FitBit, that enabled them to monitor their physical activity and sedentary time and incentives points, and watch instructional videos about the study equipment (FitBits, Wi-Fi scales, blood pressure monitors).

Calculation of incentives was based on physical activity and sitting time data assessed by the FitBit One. Prior to the intervention, a preliminary investigation into preferences for FitBit models showed that the FitBit One device (worn on a belt or in a pocket) was more often preferred by similarly-aged individuals (*n* = 21) compared to the wrist-worn FitBit Flex. The FitBit One is a valid device for measuring physical activity among free-living healthy adults [24]. During the day, participants wore the FitBit One clipped into their belt band or in their pocket; at night, they placed it into a sleep band worn on the wrist, then set it into sleep mode until they woke. Using sleep mode ensured that sedentary minutes could be determined separately from time spent sleeping.

Participants were instructed to wear their FitBit for 3 days at baseline while continuing to perform their usual levels of physical activity and sedentary behaviors, to establish a baseline sedentary time (average minutes sedentary across the 3 days) against which reductions in sitting time during the intervention period could be assessed. Manual calculation of physical activity and sedentary points was necessary for eight participants at some point in the study, for a variety of reasons. These included three participants notifying the research team of concerns that the FitBit One device provided was faulty (although no evidence was found that this was the case), and that their baseline physical activity or sedentary minutes were inaccurately captured on at least one of the three baseline days; in these cases an average of the remaining days was accepted as a baseline. In other instances, incorrect registration on the ACHIEVE website resulted in one participant not accruing points; and several other participants lost their FitBit and therefore did not record any activity while they waited for a new device to be posted. Where one participant had registered incorrectly, it was possible to retrieve FitBit data from study commencement and manually calculate physical activity and sedentary points retrospectively. In cases where participants had lost their FitBit, points for the missing few days/week were manually calculated based on averages of immediately preceding weeks.

A time was organized for the motivational interview to be conducted by telephone within the next week, at which point the intervention period began.

### Evaluation

Feasibility was considered in terms of the success of processes for recruiting and retaining participants and implementing the incentives program. Effectiveness was evaluated by comparing physical activity, sedentary behavior, adiposity and blood pressure at pre- and immediately post-intervention. FitBits were used to assess qualifying for incentives, rather than as a measure of study outcomes. Physical activity and sedentary behavior were assessed using the International Physical Activity Questionnaire (IPAQ) long format, which assesses, among other domains, time spent in walking, moderate and vigorous physical activity for transport and for leisure; and the duration of time participants spent sitting on weekdays and weekend days overall. At both time points, participants completed online surveys via Qualtrics (Qualtrics, Provo, UT). Program appeal was examined at post-intervention by self-report questions assessing participants’ agreement on a 5-point Likert scale with the statements: The weekly [text messages/motivational interview/incentives] were helpful; I liked the types of incentives offered; and the incentive points motivated me to [be more active/reduce my sitting time]. They were also asked (yes/no) if the ACHIEVE program helped them be more active/sit less.

BMI (kg/m^2^) was calculated from height (objectively measured by researchers at baseline) and weight (objectively measured by researchers at baseline, and measured by participants using Wi-Fi scales provided at post-intervention). At baseline and immediately post-intervention, participants recorded systolic and diastolic blood pressure readings on the Omron blood pressure monitor on any 3 days over the course of a week and noted these on a record sheet. Readings were taken at the same time each day, and while the participant was calm and sitting quietly. Participants were instructed not to take measurements immediately following meals or exercise, or while stressed. Participants returned the monitors and record sheets to the research team by post, at which point the research team averaged the three readings to provide measures of systolic and diastolic blood pressure.

### Statistical analyses

Paired t-tests were used to analyze changes from baseline to follow-up in all outcomes in the sample as a whole, and separately for men and women. Effect sizes for dependent groups were calculated as Cohen’s d_z_ = (t /√n) [25]. Descriptive statistics were used to summarise markers of feasibility and appeal.

## Results

### Feasibility

We easily managed to recruit and retain the required sample (*n* = 80) from our initial targeted mail-out, with only two of our original 82 participants withdrawing, demonstrating the feasibility of recruiting and retaining inactive middle-aged participants to an incentives-based physical activity/reduced sitting intervention over a 4-month period. Apart from the few instances of incorrect registration, FitBit loss, or perceived malfunction, the web-based platform worked effectively in calculating incentive points based on FitBit data. Adherence in terms of wearing and syncing Fitbits was high, with 84% of participants providing (non-zero) Fitbit data every week during the intervention. Of the remaining 16% (*n* = 13), five missed only 1 week, five missed 2 weeks, and only three missed more than 2 weeks (one of whom lost their Fitbit).

Descriptive data showing the proportion of participants achieving each incentive are shown in Table 2. While 66% of the sample qualified for the first (easiest) physical activity incentive of 100 mins/week, only 34% qualified for the final and most challenging incentive of 150 mins/week. Reduced sitting goals appeared more challenging, with 43% of participants qualifying for the first incentive, but only 20% for the last incentive. In almost all cases more men than women qualified for incentives.

**Table 2:** Proportion of participants (*n* = 82) qualifying for incentives during the ACHIEVE intervention (Weeks 1–16)

Incentive program	Men	Women	Total
	*N* = 36	*N* = 46	*N* = 82
Achieved sedentary behavior incentives %			
Incentive 1 (weeks 1 & 2)	78	57	66
Incentive 2 (weeks 3 & 4)^a^	66	59	62
Incentive 3 (weeks 4–8)^b^	57	31	43
Incentive 4 (weeks 9–12)	49	36	41
Incentive 5 (weeks 13–16)	49	22	34
Achieved sedentary behavior incentives %			
Incentive 1 (weeks 1 & 2)	39	46	43
Incentive 2 (weeks 3 & 4)^*a*^	34	33	33
Incentive 3 (weeks 4–8)^*b*^	29	24	26
Incentive 4 (weeks 9–12)	26	24	25
Incentive 5 (weeks 13–16)	26	16	20

^a^ *n* = 1 withdrawn

^b^ *n* = 2 withdrawn

### Effectiveness

Table 3 presents the mean physical activity, sitting time, BMI and blood pressure at baseline and follow-up for those providing complete data (*n* = 74). It shows that all variables changed in the expected direction. Leisure-time physical activity increased by 212.1 mins/week in men (mean ± SD; baseline = 106.7 ± 135.1 mins/week, follow-up = 318.8 ± 263.6 mins/week) and 281.6 mins/week in women (baseline = 81.4 ± 105.3 mins/week, follow-up = 363.0 ± 486.7 mins/week); and transport-related physical activity by 139.6 mins/week in men (baseline = 73.4 ± 85.8 mins/week, follow-up = 213.0 ± 223.3 mins/week) and 207.1 mins/week in women (baseline = 81.2 ± 94.9 mins/week, follow-up = 288.3 ± 371.4 mins/week). Sitting time decreased by 3.1 h/day for both sexes (men: baseline = 8.6 ± 2.6 h/day, follow-up = 5.5 ± 1.9 h/day; women: baseline = 8.4 ± 2.4 h/day, follow-up = 5.3 ± 2.1 h/day). Positive improvements were also seen through reductions in BMI by 1.3 kg/m^2^ (baseline = 30.6 ± 6.2 kg/m^2^, follow-up = 29.3 ± 5.8 kg/m^2^) and systolic blood pressure by 5.1 mmHg (baseline = 126.1 ± 16.4 mmHg, follow-up = 121.0 ± 13.2 mmHg). Effect sizes were small for blood pressure, but medium (>0.5) to large (>0.8) for all other outcome variables.

**Table 3:** ACHIEVE participants’ (*n* = 74) mean (SD) behavioral and biological outcomes at baseline and follow-up

	Men (*n* = 31)				Women (*n* = 43)				All (*n* = 74)			
Variable (mean (SD))	Baseline	Follow-up	*p*-value	ES	Baseline	Follow-up	*p*-value	ES	Baseline	Follow-up	*p*-value	ES
Leisure-time physical activity (mins/week)	106.7 (135.1)	318.8 (263.6)	<0.001	0.83	81.4 (105.3)	363.0 (486.7)	<0.001	0.56	92.0 (118.5)	344.5 (406.6)	<0.001	0.61
Transport-related physical activity (mins/week)	73.4 (85.8)	213.0 (223.3)	0.001	0.63	81.2 (94.9)	288.3 (371.4)	<0.001	0.55	77.9 (90.7)	256.8 (318.2)	<0.001	0.56
Sitting (hours/day)	8.6 (2.6)	5.5 (1.9)	<0.001	1.13	8.4 (2.4)	5.3 (2.1)	<0.001	1.61	8.5 (2.5)	5.4 (2.0)	<0.001	1.35
BMI (kg/m^2^)	30.9 (4.9)	29.7 (4.4)	<0.001	1.14	30.3 (7.0)	29.0 (6.7)	<0.001	1.19	30.6 (6.2)	29.3 (5.8)	<0.001	1.17
Systolic blood pressure	129.8 (17.6)	123.6 (12.0)	0.008	0.51	123.5 (15.2)	119.1 (13.9)	0.005	0.45	126.1 (16.4)	121.0 (13.2)	<0.001	0.48
Diastolic blood pressure	81.8 (11.3)	78.2 (10.4)	0.017	0.45	76.4 (11.0)	75.1 (8.7)	0.258	0.17	78.7 (11.4)	76.4 (9.5)	0.013	0.29

*Abbreviations*: *ES* effect size (Cohen’s d_z_)

*p*-values based on paired *t*-tests

### Appeal

Overall, 96% of participants reported that the ACHIEVE program made a difference to their physical activity levels; 65% reported that it made a difference to their sitting time. Participants’ experiences with different aspects of the program are summarized descriptively in Table 4. The majority of the sample agreed that most intervention components were helpful, though this was lowest (50% agreeing/strongly agreeing) for the statement that incentives motivated participants to reduce their sitting time.

**Table 4:** Proportion of the sample (*n* = 80) endorsing process evaluation statements about the ACHIEVE program

Process evaluation statement	Proportion (%) endorsing each response category				
	Strongly Agree	Agree	Neither agree nor disagree	Disagree	Strongly disagree
The weekly text messages were helpful	10.8	50.0	20.3	16.2	2.7
The motivational interview was helpful	31.1	54.0	13.5	1.4	0
The incentives were helpful	32.4	40.5	10.8	12.2	4.1
I liked the types of incentives offered	20.3	48.7	25.7	4.0	1.3
The incentive points motivated me to be more active	24.3	33.8	24.3	14.9	2.7
The incentive points motivated me to reduce my sitting time	25.7	24.3	29.7	18.9	1.4

## Discussion

Results of this pre-post-test design trial showed that the ACHIEVE incentives-based program produced promising results in terms of feasibility, appeal and effectiveness. It not only improved the target behaviors, physical activity and sitting time; it also impacted these behaviors with a sufficient intensity to effect favorable changes on key health outcomes, BMI and blood pressure. The observed effect sizes are substantial, particularly when compared with those of the few existing incentive-based physical activity studies. For instance, in a recent meta-analysis of incentives-based approaches for promoting health behaviors [7], only three studies assessed physical activity, and none of these found significant effects of financial incentives on activity levels. The effects observed here also compare well against those obtained in meta-analyses of other intervention approaches to promote physical activity, which have reported mean pre-post treatment effect sizes of 0.33, equating to around 25 mins/week additional physical activity [26].

The fact we so quickly recruited the sample suggests high interest in an intervention of this nature, reinforcing the idea that incentives have a key role to play in initiation of a behavior. Indeed, individuals encounter incentives-based approaches in everyday life, for example through loyalty schemes used by many commercial organisations to attract and retain customers, or to promote purchasing of particular goods or services. While incentive programs clearly garner interest in the short-term, their longer-term impact on behavior change is not known. Older studies on reinforcement of behavior that shows that whilst rewards may be effective at changing behavior, the effects are unlikely to be maintained when the rewards are withdrawn in the absence of other interventions [27]. This is why we embedded the incentives into a more comprehensive approach. The program was designed to enhance both intrinsic, as well as the initial extrinsic motivation that might be provided by the incentives. Such an approach may help minimize the risk of relapse through at least two possible mechanisms. Firstly, enhancing skills, behavioral techniques and self-efficacy for increased physical activity and reduced sitting behaviors may help to make these behaviors habitual by the time the rewards are withdrawn, consistent with automaticity theories [28]. Secondly, participants may feel satisfied with the benefits of the increased activity/decreased sedentariness (e.g., weight loss, feeling fitter/better), and so wish to maintain it to continue to receive these benefits, consistent with Rothman's theory of maintenance [29]. Future incentives-based studies could test these theories.

The goals linked to incentives were designed to be challenging but not impossible to achieve. Results suggest this was the case, with some, but not all participants qualifying for incentives. Generally more men than women achieved incentives, which is consistent with evidence that men tend to be more likely to be active and less likely to be sedentary than women [16]. The sedentary behavior goals were generally achieved by fewer participants, and descriptive data on program appeal suggested the program was less helpful for reducing sitting time than for increasing physical activity. It may be that the sedentary behavior goals were not appropriate for the participants. Kremers et al. [30] have proposed there may be automatic and unconscious influences on behaviors like television viewing, which is one of the most common sedentary behaviors amongst adults. Watching television is a routine behavior that is repeatedly performed and likely to be determined by habit [30]. Because it may be automatic, breaking a habit like television viewing may therefore be more challenging that initiating a new behavior, such as increasing physical activity. Incentives may need to be supplemented in concert with additional strategies specifically targeting sedentary behaviors, such as more intensive support for reducing or breaking up television viewing time, or workplace programs to reduce sitting time at work.

The evidence base is currently too limited to enable conclusions as to which components might make an incentives-based approach most effective, and for whom. The current results add some insights into particular promising attributes. Based on the limited available evidence (e.g., Mitchell et al. [6]), we targeted inactive participants, and employed an incentives schedule that was escalating, periodic, with assured incentives (in all but one case), valued from $7.50 to $50 (apart from the iPad draw), provided soon after qualifying. The increasing level of challenge seemed reasonable, with most meeting early incentives, and fewer meeting subsequent more challenging incentives. Embedding incentives into a broader program appeared appealing, with most participants reporting finding not just the incentives, but also the motivational interview and text messages helpful. Examining the impact of varying the value, timing, and challenge required to achieve incentives in future studies could provide additional insights for tailoring future programs. Similarly, further insights into how incentives are framed would be valuable, with some evidence that ‘loss-framed’ approaches (e.g., in which participants lose an incentive from an initial ‘deposit’ for each occasion they do not meet a physical activity goal) may be even more effective than the gain-framed approach trialed here.

Despite their promise, the use of incentives to encourage behavior change has been criticized, with concerns that such approaches could be stigmatizing, coercive or construed as a ‘bribe’; discriminate against those who already engage in the target behavior, or those who cannot comply; and fail to appropriately consider broader contextual influences on behavior [31, 32]. However, evidence demonstrates that financial incentives tend to be acceptable to the public when they are effective, cost-effective, and provided alongside health education and behavior change support [31, 33]. The cost-effectiveness of the ACHIEVE program will be reported in a future paper.

### Strengths and limitations

Limitations of this study include the uncontrolled design, although pre-post-test designs represent a useful preliminary method for establishing feasibility and potential effectiveness prior to launching a more costly randomized controlled trial. The study duration was short and future trials should examine longer-term maintenance of effects. Outcome measures were self-reported, although we provided both Bluetooth weighing scales and blood pressure monitors for participants to obtain those measures at follow-up. While the IPAQ measure of physical activity and sitting time is validated, some over-reporting may have occurred. For example, average IPAQ-reported combined leisure and transport physical activity minutes at baseline exceeded the recommended 30 mins/day, despite participants self-screening as not meeting physical activity guidelines; and the magnitudes of changes in both physical activity and sitting time were larger than those reported in other behavioral interventions [26]. Future studies could use objective measures of physical activity and sitting time, such as accelerometers and inclinometers. Since participants were interviewed when available across the working week, the three baseline data collection days were not consistent, and may have included weekend days for some participants but not others. Future studies might standardize this, given potential variations in sitting/activity across workdays/weekends which might impact baseline estimates and make it easier for some participants to accrue points relative to baseline than others. The use of the GMHBA database may have resulted in the recruitment of a more affluent sample (although income was not assessed given this was not a key outcome of focus), although GMHBA membership is sociodemographically diverse. On the other hand, there is some evidence that the effects of incentives may be even stronger amongst those experiencing socioeconomic disadvantage [7]. The use of the GMHBA database also provided an opportunity to link intervention participation to health claims data in a future study to examine intervention effects on health service use. Other strengths of the study include the objective assessment (FitBit) that formed the basis of establishing study fidelity and achievement of incentives, although the potential impact of the FitBit as a motivational tool alongside the incentives and other intervention components requires evaluation in future studies. A further strength was the comprehensive theory-based intervention approach accompanying the incentives, to enhance intrinsic motivation.

## Conclusions

This study showed that an incentives-based program, enhanced with behavior change support, was feasible to implement, appealing to participants, and led to significant positive effects on both physical activity and sedentary behaviors, as well as improvements in BMI and blood pressure. The program warrants future investigation in a controlled trial over a longer duration, to establish effects on sustained behavior change and associated health outcomes.

## Abbreviations

ACHIEVE Study: The Active Choices IncEntiVE Study
BMI: Body mass index
IPAQ: International Physical Activity Questionnaire
